# Research on the influencing factors of adult learners' intent to use online education platforms based on expectation confirmation theory

**DOI:** 10.1038/s41598-024-63747-9

**Published:** 2024-06-04

**Authors:** Guoqiang Pan, Yu Mao, Ziyuan Song, Hui Nie

**Affiliations:** 1 College of Continuing Education, Shanghai Normal University, Shanghai, China; 2 School of Humanities, Shanghai Normal University, Shanghai, China; 3 School of Education, Shanghai Normal University, Shanghai, China; 4 School of Foreign Language Studies, Shanghai University of Engineering Science, Shanghai, China

**Keywords:** Adult learners, Online education platform, Expectation confirmation model, Expectation confirmation, Continuance intention, Psychology, Human behaviour

## Abstract

This study addresses the understanding gap concerning the factors that influence the continuous learning intention of adult learners on online education platforms. The uniqueness and significance of this study stem from its dual focus on both platform features, such as service quality, and course features, including perceived interactivity and added value, aspects often overlooked in previous research. Rooted in Expectation Confirmation Theory, the study constructs a comprehensive model to shed light on the complex interplay of these factors. Empirical evidence collected from a survey of 1592 adult learners robustly validates the effectiveness of this model. The findings of the study reveal that platform service quality, perceived interactivity, and perceived added value significantly amplify adult learners' expectation confirmation and perceived usefulness. These elements subsequently enhance learner satisfaction, fostering their ongoing intention to use online education platforms. These insights offer practical guidance for online education providers, emphasizing the necessity to enhance platform service quality and course features to meet adult learners' expectations and perceived usefulness. The study provides valuable perspectives for devising strategies to boost user satisfaction and stimulate continuous usage intention among adult learners in the intensely competitive online education market. This study enriches the literature by uncovering the relationships among platform features, course features, expectation confirmation, perceived usefulness, and continuous usage intention. By proposing a comprehensive model, this study provides a novel theoretical basis for understanding how platform and course features impact adult learners' ongoing intention to use online education platforms, thereby aiding the evolution and refinement of relevant theories.

## Introduction

In the beginning, adult higher education primarily relied on face-to-face instruction, utilizing weekends and evenings for classes. However, frequent issues arising from the pandemic, pronounced contradictions in adult learning, and uneven distribution of time and energy led to subpar teaching quality and low efficiency^1,2^. Consequently, the introduction of online education platforms for blended learning became necessary.

Adult learners exhibit a range of learning goals and motivations^3^ while concurrently grappling with the constraints of time and space^4,5^. These variables significantly impact their inclination towards utilizing online education platforms. Unlike traditional students, adult learners often have a wider spectrum of learning objectives^6^, encompassing facets such as career progression, personal interests, and lifelong learning^7–9^. They also demonstrate stronger learning motivation and autonomy, underscoring the importance of personalized learning experiences and resources^10^. The learning outcomes for adult learners on online education platforms are influenced by factors such as course design, the richness of learning resources, and the selection of learning paths^11,12^.

For online education platforms, the associated costs of retaining existing users generally surpass those of acquiring new ones^13^. Since online education platforms function as a type of information system, researchers typically probe user behavior within these systems using technology acceptance models and expectancy confirmation models. When investigating users' continued usage intentions, most scholars extend these models based on the expectancy confirmation model. However, the front-end influencing factors for the key variables of expectancy confirmation and perceived usefulness have not been extensively studied. Currently, the factors influencing perceived usefulness are mainly examined from viewpoints such as perceived ease of use, content-driven factors, social influence, subjective norms, and autonomy^14–18^. Meanwhile, the factors affecting expectancy confirmation are explored from perspectives such as perceived playfulness, information quality, and service quality^19–21^. Nonetheless, there is a dearth of comprehensive investigation into the influencing factors of perceived usefulness and expectancy confirmation from the broad perspective of online education platform constituents, namely considering technological platform features and online course features. This gap in systematic analysis impedes the ability of online education platforms to adopt effective service improvement measures aligned with their unique characteristics.

In light of the distinct attributes of adult learners participating in online education, this research conducts a systematic review and a thorough consolidation of the determinants impacting perceived usefulness and expectation confirmation^8^. This is done from the standpoint of both technological platform characteristics and the attributes of online courses^9^. By adopting such a comprehensive approach, we can more effectively aid online education platforms in identifying and implementing service improvement measures that resonate with their unique traits.

This study concentrates on online education platforms and adult learners, examining the potential influence of platform features (such as platform service quality) and course attributes (like perceived interactivity and the perceived added value of the course) on adult learners' perceived usefulness and expectation confirmation when engaging with online education platforms. Furthermore, this study delves into the effects of these factors on adult learners' satisfaction and their continued intention to use these platforms.

In the context of online education platforms, the diverse learning objectives and motivations of adult learners^2^, coupled with the constraints they encounter in terms of time and space^5^, play a crucial role. These elements shape their willingness to engage with online education platforms. In contrast to traditional students, adult learners may have a broader range of learning goals, encompassing career advancement, personal interests, and lifelong learning^12^. They exhibit a heightened sense of learning motivation and autonomy, placing a premium on personalized learning experiences and resources^9^. Furthermore, online education platforms can impact the learning outcomes of adult learners through factors such as course design, the abundance of learning resources, and the selection of learning paths^8,22^. Consequently, our research will concentrate on how these factors affect the learning outcomes of adult learners on online education platforms, aiding us in better comprehending and catering to their learning requirements.

## Theoretical foundation and research hypotheses

### Expectation confirmation theory

In 1980, Oliver introduced the Expectancy Disconfirmation Theory (EDT). This theory posits that users, prior to purchasing a product or service, hold certain expectations. After the actual use of the product or service, users perceive the performance differential between their expectations and the realized experience, termed as expectancy disconfirmation^23^.

The Expectancy Confirmation Theory (ECT) has evolved from the Expectancy Disconfirmation Theory (EDT) and serves as a crucial foundation for studying user continuance. Patterson et al. were among the pioneers to apply the Expectancy Confirmation Theory in the field of information systems^24^. Bhattacherjee proposed the Expectation Confirmation Model (ECM-ISC), which incorporates four main variables: expectation confirmation, perceived usefulness, satisfaction, and continuance intention^25^. Following the introduction of the Expectation Confirmation Model, studies by Larsen on mobile commerce^26^, Tang and others on blogs^27^, Doong on knowledge sharing^28^, and Kim on mobile data services^29^ have all affirmed the effectiveness of the Expectation Confirmation Model.

### Research hypotheses

In the online education environment for adult learners, platform features refer to the key factors influencing satisfaction and exert a significant impact on adult learners' continued usage intentions. Among these features, platform service quality is crucial and measures the effectiveness and promptness of services provided by service providers. Bhattacherjee defines expectancy confirmation as the degree to which information system users confirm their expectations before and after using the system, where lower expectations and higher actual experiences enhance expectancy confirmation^25^. Researchers such as Dahan et al. have validated through data the positive impact of service quality on expectancy confirmation and satisfaction^30^. Delone et al. through the Information Success Model they constructed, identified service quality as a critical factor influencing satisfaction and usefulness^31^. Moreover, existing research has confirmed the significant influence of service quality on perceived usefulness and expectancy confirmation^27,32,33^.

Adult learners in online education place a greater emphasis on personalized learning experiences and resources, making the service quality of the platform crucial to their actual learning experiences^13,34^. Based on this, we hypothesize that the higher the platform's service quality, the better the actual learning experience for adult learners. Based on this, the following research hypotheses are proposed:

**H1:** Platform service quality positively influences the expectancy confirmation of adult learners.

**H2:** Platform service quality positively influences the perceived usefulness of adult learners.

In online education platforms, in addition to platform features, course characteristics are considered the most crucial factors for adult learners' attention^35^. This study measures course features through perceived interactivity and perceived added value. The more satisfied adult learners are with course features, the stronger their intention to continue using the platform. In traditional educational settings, interactions with teachers and peers positively influence students, and similar positive effects are expected in online education. Emphasizing platform interactivity facilitates communication among adult learners, timely issue resolution, and enhances their expectations^36^. If the platform lacks strong interactivity, it may negatively impact the learning experience^37^. Therefore, platforms need to enhance interactivity to cultivate positive online learning habits among adult learners^38^. The stronger the perceived course features, the higher the adult learners' overall satisfaction with the course experience. Yang's study on MOOC users found that interactivity significantly influences expectancy confirmation^20^. Perceived added value refers to additional or value-added services provided beyond basic services^39,40^. As an additional benefit, it further enhances users' perceived value of the course, leading to a more positive evaluation of expectancy confirmation. Online education platforms should focus on improving course features, enhancing interactivity, and providing perceived added value to better meet the needs of adult learners^41^. Based on the characteristics of adult learners in online education, this study proposes the following hypotheses:

**H3:** Course characteristics significantly influence the expectancy confirmation of adult learners.

**H3-1:** Perceived interactivity has a significant positive impact on the expectancy confirmation of adult learners.

**H3-2:** Perceived added value has a significant positive impact on the expectancy confirmation of adult learners.

Based on data from the SPOC platform, Guo et al. found that classroom interaction significantly and positively influences perceived usefulness and perceived ease of use in SPOC learning^42^. A research of Wu et al. indicated that interactivity has an impact on perceived usefulness and expectancy confirmation^43^. The study of Qian et al. revealed that perceived interactivity has a positive effect on perceived usefulness^15^. Additionally, Yang found a positive influence of interactivity on perceived usefulness in research involving MOOC users^20^. In the field of mobile communication services, Liu and Chen examined the impact of added value on perceived usefulness and discovered that value-added services have a positive effect on the perceived usefulness and satisfaction of communication customers^44^. This finding aligns with the personalized learning experience needs of adult learners. Based on these observations, the following research hypotheses are proposed:

**H4:** Course features have a significant impact on the perceived usefulness of adult learners.

**H4-1:** Perceived interactivity has a significantly positive influence on the perceived usefulness of adult learners.

**H4-2:** Perceived added value has a significantly positive impact on the perceived usefulness of adult learners.

Bhattacherjee validated the impact of expectancy confirmation on perceived usefulness in the Expectation Confirmation Model^25^. Perceived usefulness refers to the improvement in learning efficiency and the degree of learning outcomes when users utilize online education platforms. Perceived usefulness not only influences adult learners' initial acceptance^45^ but also has a significant impact on adult learners' satisfaction and the intention to continue using the platform^25^. Yang, in his study on MOOC users, confirmed the positive effect of expectancy confirmation on perceived usefulness^20^. Qian's research on online learning users also affirmed the positive influence of expectancy confirmation on perceived usefulness^15^. Studies by Hayashi and Lin further supported the impact of expectancy confirmation on perceived usefulness^46,47^. Based on this, the following research hypothesis is proposed:

**H5:** Expectation confirmation positively influences the perceived usefulness of adult learners.

When adult learners' expectations are met or exceeded by the online education platform, signifying a higher level of expectancy confirmation, it leads to increased satisfaction with the platform. This hypothesis is supported by research done on individual users in Social Networking Sites (SNS) as well as studies conducted by Liu and colleagues on short video users^48^. Further, the findings of Chiu et al. and Wang et al. reinforce the influence of expectancy confirmation on satisfaction^49,50^. Moreover, this relationship has been substantiated in various digital contexts, such as e-commerce^51^, and e-learning^52^, suggesting that expectancy confirmation is a significant predictor of user satisfaction across different digital platforms. To further expand on this, it's worth noting that expectancy confirmation can also influence other aspects of user experience. For instance, when users' expectations are confirmed, they may perceive the platform as more useful, which can further enhance their satisfaction^53^. Additionally, expectancy confirmation can also impact users' trust in the platform^9^. When users' expectations are met, they may develop a higher level of trust in the platform, which can also contribute to increased satisfaction. Based on this, the following research hypothesis is proposed:

**H6:** Expectancy confirmation positively influences user satisfaction with the online education platform.

Wang et al. affirmed the positive influence of perceived usefulness on satisfaction in their study of users of Virtual Reality (VR) library services^54^. In a similar vein, Yin et al.'s research on WeChat users in university libraries corroborated the positive effect of perceived usefulness on satisfaction^55^. The connection between perceived usefulness and satisfaction was further explored and substantiated in studies by Bhattacherjee and Lin et al.^25,47^. These findings collectively underscore the importance of perceived usefulness in driving user satisfaction across a variety of digital platforms. Building on these insights, we can argue that perceived usefulness is not just an antecedent of satisfaction, but may also play a role in shaping other user attitudes and behaviors^56^. For example, perceived usefulness could influence users' continued intention to use a platform^52^, their trust in the platform^57^, and their willingness to recommend the platform to others^58^. Based on these findings, the following research hypothesis is proposed:

**H7:** Perceived usefulness positively influences the satisfaction of adult learners.

This study focuses on the intention to continue use, which refers to adult learners' willingness to continue using online education platforms. Satisfaction is the experiential feeling and overall evaluation that adult learners have after using online education platforms. Cao et al., focusing on WeChat Moments experience, found that satisfaction significantly influences users' intention to continue using^59^. The research of Zhang and Yao on mobile government apps also confirmed the impact of satisfaction on the intention to continue use^60^. Scholars such as Bhattacherjee et al., have all verified the positive influence of satisfaction on the intention to continue use^20,25,46^. Based on this, the following research hypothesis is proposed:

**H8:** Satisfaction positively influences the intention to continue use of adult learners.

The extent of users' intention to continue using online education platforms reflects their loyalty to the selected platform. The study of Gao and Hu on users of knowledge community services found that service quality has a positive impact on continued usage^61^. Lin, through research on consumer behaviors using the ABC attitude theory, discovered that service quality positively influences shopping attitudes^62^. Zhou et al., in their study of users in the shopping domain, identified service quality as a significant influencing factor on intention to continue usage^63^.

The influence of platform service quality on the intention to continue use among adult learners may be subject to the mediating effects of other variables. Wang's study on the continued use intention of mobile libraries found that service quality affects users' perceived usefulness^64^. Guo and Ming concluded that service quality positively influences users' expectation confirmation and perceived usefulness^65^. Hsu and Lin discovered in their study of mobile client user behavior that service quality initially affects user satisfaction^66^. Yang also identified service quality as a significant factor influencing user satisfaction in mobile reading^67^. Alali and Salim, in their study on a health forum, found a significant impact of service quality on user satisfaction^68^. In the field of information systems, scholars have confirmed the positive impact of expectation confirmation on user satisfaction^46,47,69,70^. This implies that when users have high expectation confirmation, indicating their expectations and usage experience are satisfied, it can enhance their perceived usefulness and increase satisfaction with the platform. Liu's research on video website users validated the effect of perceived usefulness on satisfaction^71^. Yang's study on e-book users also confirmed the positive impact of perceived usefulness on satisfaction^72^. Concurrently, within the realm of consumer behavior, a multitude of empirical investigations have corroborated the affirmative promotional influence of satisfaction on users' proclivity to sustain usage^15,20,25,67^. This suggests that expectation confirmation has an impact on adult learners' satisfaction, and learner satisfaction may further influence their intention to continue use. Combining the positive influence relationship of platform service quality on adult learners' expectation confirmation and perceived usefulness proposed in H1 and H2, this study posits that platform service quality has a positive impact on the intention to continue use among adult learners. Moreover, this positive influence occurs through multiple mediating effects of perceived usefulness, expectation confirmation, and satisfaction among adult learners. Based on this, the following research hypothesis is proposed:

**H9:** Platform service quality has a positive impact on the intention to continue use of adult learners.

**H9-1:** In the process of the impact of platform service quality on the intention to continue use of adult learners, expectation confirmation and satisfaction play a chain-mediating role.

**H9-2:** In the process of the impact of platform service quality on the intention to continue use of adult learners, perceived usefulness and satisfaction play a chain-mediating role.

In terms of the impact of course features on adult learners' intention to use, researchers have made some important findings. The study of Joo et al. discovered that high-quality interactions can stimulate positive evaluations of educational platforms by users, thereby increasing their intention to continue using^73^. This indicates that high-quality interactions, such as timely answering of questions and sharing ideas, can enhance adult learners' loyalty to the platform. Chow et al. investigated the impact of interactions on users' intention to continue using from the dimensions of teacher interaction and peer interaction^74^. The research of Hoffman and Novak found that the higher the user's interactivity, the better their overall experience^75^. In addition, Zhang and Wu pointed out that perceived interactivity can lead to positive emotional changes in users^76^. Perceived added value is the unexpected gain that adult learners feel, which can effectively increase their favorability towards the platform and satisfaction with the usage process^42^, thereby reinforcing their intention to continue using.

Van Noort et al. found that the higher users' perceived interactivity on a website, the higher their satisfaction and willingness to use the website^77^. Qian validated the positive impact of perceived interactivity on satisfaction and continued intention to use^15^. Gefen et al. argued that user interaction with a website can influence their trust attitudes^78^. Kim's study on travel websites revealed that interactivity can enhance users' trust in the website^79^. Park et al. focused on the impact of perceived interactivity on satisfaction and examined the mediating role of perceived usefulness/perceived value^80^. Song and Zinkhan found that website response speed is a crucial factor influencing user satisfaction^81^. Meanwhile, in the field of information systems, numerous scholars have confirmed the influence of expectation confirmation and perceived usefulness on satisfaction, as well as the impact of satisfaction on intention to continue using^15,20,25,67^. Combining with the proposed positive relationships in H3 and H4 regarding course features (perceived interactivity and perceived added value) and adult learners' expectation confirmation and perceived usefulness, this paper suggests that online education course features have a positive impact on adult learners' intention to continue using. Moreover, this positive influence occurs through multiple mediating pathways involving adult learners' perceived usefulness, expectation confirmation, and satisfaction. Based on this, the following research hypotheses are proposed:

**H10:** Course features significantly impact adult learners' intention to continue using.

**H10-1:** We posit that perceived interactivity has a positive influence on the continued use intention of adult learners. In the process of how perceived interactivity influences the intention to continue use, we propose that both expectation confirmation and satisfaction, as well as perceived usefulness and satisfaction, play a chain-mediating role. This suggests that when the perceived interactivity of the course meets or exceeds the expectations of adult learners, it confirms their expectations, enhances their satisfaction, and simultaneously elevates the perceived usefulness of the course, ultimately influencing their intention to continue using the platform.

**H10-2:** We suggest that perceived added value has a positive influence on the continued use intention of adult learners. In the process of how perceived added value influences the intention to continue use, we propose that both expectation confirmation and satisfaction, as well as perceived usefulness and satisfaction, play a chain-mediating role. This implies that when the perceived added value of the course meets or exceeds the expectations of adult learners, it confirms their expectations, enhances their satisfaction, and simultaneously increases the perceived usefulness of the course, ultimately influencing their intention to continue using the platform.

**Figure 1:**
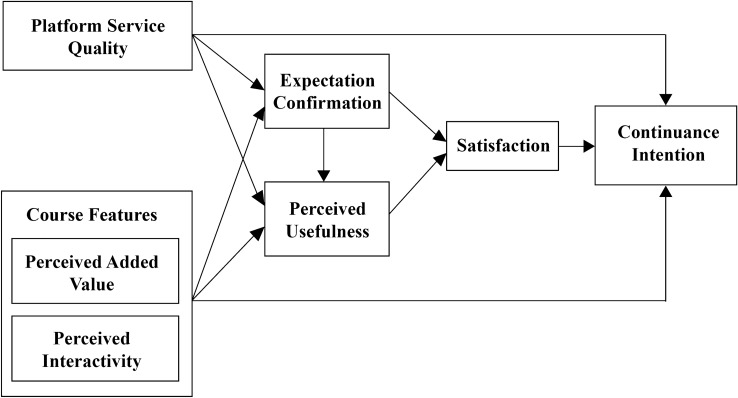
Research model framework.

In summary, the research model of factors influencing the intention to continue using online education platforms in this study is illustrated in Fig. 1.

## Method

### Measurement tools

The design of the questionnaire drew inspiration from mature scales used globally to measure the intention to use information systems. Additionally, references were taken from relevant studies on online education platforms both domestically and internationally, with subsequent modifications made to the questionnaire. The questionnaire comprises two parts: the first part gathers adult learners demographic information, while the second part measures the factors influencing the continuous usage behaviour of online education platform adult learners (refer to Table 1). The Likert five-point scale method was employed in the questionnaire, where 1 represents *strongly* *disagree*, 2 represents *disagree*, 3 represents *neutral*, 4 represents *agree*, *a*nd 5 represents *strongly* *agree*.

**Table 1:** Measurement items and reference sources.

Variable	Measurement indicators	Reference source
Expectation confirmation	EC1: My experience with using the online education platform has been better than I expected	^25,82^
	EC2: The services and features provided by the online education platform have been better than I expected	
	EC3: Overall, my expectations for learning through the online education platform have been met	
Perceived usefulness	PU1: Learning through the online education platform can enhance my learning outcomes	^25,83^
	PU2: Learning through the online education platform can improve my learning efficiency	
Satisfaction	SA1: I am satisfied with the decision to learn via the online education platform	^25,84^
	SA2: I am satisfied with the outcomes of learning through the online education platform	
	SA3: I am satisfied with my overall experience of using the online education platform	
Continuance intention	CI1: I am willing to continue using the online education platform for learning	^82,85^
	CI2: I am willing to use the online education platform regularly for learning in the future	
	CI3: I am willing to increase the frequency of using the online education platform for learning	
Perceived interactivity	PI1: When learning through the online education platform, I can interact effectively with teachers and classmates	^86–88^
	PI2: When learning through the online education platform, other students and I can communicate and discuss in a timely manner	
	PI3: When learning through the online education platform, course instructors can answer my questions promptly	
Perceived added value	PAV1: The online education platform provides unexpected complimentary services to students	^89^
	PAV2: The online education platform provides unexpected supplementary course materials to students	
	PAV3: The online education platform provides complimentary resources beyond the course content	
Platform service quality	PSQ1: The customer service of the online education platform can respond to my questions promptly	^31,90^
	PSQ2: The customer service of the online education platform can provide reliable and accurate answers to my questions	
	PSQ3: The online education platform can offer professional service support	

### Participants

This study selected adult education students from a university in Shanghai as research subjects. By employing a stratified sampling method, we selected participants based on 10% of the total adult student population. This sampling process was carried out stratifying by profession and grade. The total number of participants amounted to 1592, the detailed information of which can be found in Table 2. Prior to participants answering the questionnaire, there was an introductory statement informing them of the purpose of the survey, emphasizing the confidentiality, anonymity, and voluntary nature of their participation in the research.

**Table 2:** Demographic information of participants (N = 1592).

Characteristic	Range	Frequency	Percentage
Gender	Male	922	57.91
	Female	670	42.09
Age	21–30 years	554	34.80
	31–40 years	580	36.43
	41–50 years	266	16.71
	51–60 years	157	9.86
	60 years or more	35	2.20
Employment status	Full-time	989	62.12
	Part-time or temporary work	350	21.98
	Freelance	166	10.43
	Unemployed	87	5.46
Work tenure	Less than 3 years	372	23.37
	4–6 years	576	36.18
	7–9 years	299	18.78
	10–12 years	199	12.50
	13–15 years	102	6.41
	16 years or more	44	2.76

### Data processing methodology

We used SPSS 23.0 software to first test for common method bias in the data and analyse the correlations between variables. AMOS 24.0 software was employed to test the discriminant validity among variables. Additionally, SPSS 23.0 software and the PROCESS 2.16 macro program with the Bootstrap test method (setting the sample extraction size to 5000 times and the confidence interval to 95%) were used to test for the chained mediation effects.

### Informed consent statement

We confirm that informed consent has been obtained from all subjects. Each survey will provide an Informed Consent Form, which will be indicated in the instruction section of the questionnaire.

## Results

### Common method bias

To avoid the potential issue of substantial common method bias influencing the spurious prediction of independent variables on dependent variables, this study employed two methods (procedural control and statistical control) for control and examination^91^.

Firstly, before distributing the survey questionnaire, this study implemented effective randomization of the various scales used and made a commitment to participants to protect the privacy of their data. Secondly, the study employed the Harman's single-factor test for statistical control. Through exploratory factor analysis conducted on the obtained data without factor rotation, the first factor's variance explained 39.26% (below the critical threshold of 40% variance explained)^92^. Therefore, these methods and results suggest that there is no severe common method bias in the collected data for this study.

### Reliability and validity analysis

Through the application of SPSS 23.0, an examination of the reliability and validity of the questionnaire was conducted. The Cronbach's alpha value for the questionnaire was found to be 0.896. Furthermore, the Cronbach's alpha values for each variable were all above 0.689 (refer to Table 3), indicating a good level of internal consistency for the variables. Hence, the reliability of the questionnaire is deemed acceptable. The Kaiser–Meyer–Olkin (KMO) value for the questionnaire was 0.914, with individual variable KMO values exceeding 0.5. The overall interpretability is high, justifying the application of principal component analysis. The cumulative variance explanation rate was determined to be 67.779%. Moreover, all factor loading values were above 0.5, signifying good validity of the sample. These results affirm the reliability and validity of the questionnaire, ensuring the robustness of the data analysis and interpretation^93–97^.

**Table 3:** Variable indicators and factor analysis results (N = 1592).

Variable	Item	Cronbach's α	Factor loading
Expectation confirmation	EC1	0.755	0.789
	EC2		0.742
	EC3		0.733
Perceived usefulness	PU1	0.724	0.766
	PU2		0.713
Satisfaction	SA1	0.733	0.723
	SA2		0.743
	SA3		0.702
Continuance intention	CI1	0.693	0.738
	CI2		0.687
	CI3		0.690
Perceived interactivity	PI1	0.783	0.815
	PI2		0.763
	PI3		0.656
Perceived added value	PAV1	0.689	0.743
	PAV2		0.686
	PAV3		0.683
Platform service quality	PSQ1	0.726	0.733
	PSQ2		0.712
	PSQ3		0.705

### Fit test analysis

The purpose of model fit testing is to measure the degree of fit between the hypothetical model and the observed data. As shown in Table 4, overall, the research model exhibits good fit.

**Table 4:** Model fit analysis (N = 1592).

Goodness-of-fit index	*X*^2^/df	GFI	NFI	CFI	TLI
Results	2.125	0.956	0.913	0.965	0.961
Evaluation criteria	< 5	> 0.9	> 0.9	> 0.9	> 0.9

### Mediation effect testing

Firstly, a preliminary examination of the mediation effect was conducted using the linear hierarchical regression method. The variables showed a correlation, with coefficients between 0.4 and 0.76, and all Composite Reliability (CR) above 0.675, and all Average Variance Extracted (AVE) above 0.5 indicating no severe collinearity among the variables. The correlation results are presented in Table 5^94–97^.

**Table 5:** Descriptive statistical analysis (N = 1592).

Variables	M	SD	CR	AVE	1	2	3	4	5	6	7
1 EC	3.95	0.68	0.80	0.57	0.732						
2 PU	3.93	0.82	0.71	0.55	0.654**	0.754					
3 SA	3.88	0.76	0.77	0.52	0.436**	0.415**	0.612				
4 CI	4.12	0.79	0.75	0.50	0.453**	0.496**	0.522**	0.651			
5 PI	3.45	0.99	0.79	0.60	0.423**	0.413**	0.495**	0.533**	0.741		
6 PAV	3.73	0.86	0.75	0.50	0.678**	0.658**	0.412**	0.514**	0.499**	0.625	
7 PSQ	3.85	0.83	0.76	0.51	0.518**	0.504**	0.402**	0.454**	0.432**	0.564**	0.687

*EC* Confirmation of expectations, *PU* perceived usefulness, *SA* satisfaction, *CI* continuance intention, *PI* perceived interactivity, *PAV* perceived added value, *PSQ* platform service quality, The diagonal bold word is square root of average variance extracted (AVE). *p < 0.05, **p < 0.01, ***p < 0.001.

Furthermore, the Bootstrap method, as implemented in the Process macro program with 5000 resamples and a confidence interval set at 95%, was employed to conduct a more in-depth examination of the chain mediation^46^.

**Figure 2:**
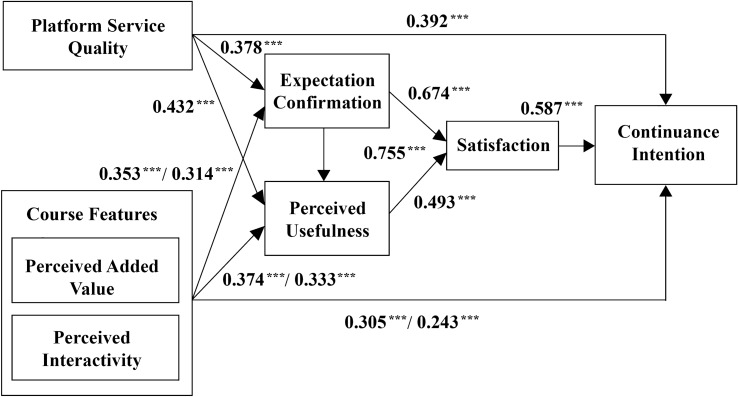
Results of the regression analysis on the continued usage intention of adult learners.

From Fig. 2 and Table 6, it can be observed that platform service quality significantly influences adult learners' perceived usefulness (*β* = 0.378, *p* < 0.0001) and expectation confirmation (*β* = 0.432, *p* < 0.0001), supporting hypotheses ***H1*** and ***H2***. Adult learners' perceived interactivity (*β* = 0.282, *p* < 0.0001) and perceived additional value (*β* = 0.353, *p* < 0.0001) significantly positively impact their expectation confirmation, supporting hypothesis ***H3***. Adult learners' perceived interactivity (*β* = 0.333, *p* < 0.0001) and perceived additional value (*β* = 0.374, *p* < 0.0001) significantly positively influence their perceived usefulness, supporting hypothesis ***H4***. Adult learners' expectation confirmation positively influences their perceived usefulness (*β* = 0.755, *p* < 0.0001), supporting hypothesis ***H5***. Adult learners' expectation confirmation positively influences their satisfaction (*β* = 0.374, *p* < 0.0001), supporting hypothesis ***H6***. Adult learners' perceived usefulness positively influences their satisfaction (*β* = 0.493, *p* < 0.0001), supporting hypothesis ***H7***. Adult learners' satisfaction positively influences their intention to continue using the platform (*β* = 0.587, *p* < 0.0001), supporting hypothesis ***H8***. Platform service quality significantly influences adult learners' intention to continue using (*β* = 0.374, *p* < 0.0001), supporting hypothesis ***H9***. Adult learners' perceived interactivity (*β* = 0.243, *p* < 0.0001) and perceived additional value (*β* = 0.305, *p* < 0.0001) positively influence their intention to continue using, supporting hypothesis ***H10***. All ten research hypotheses derived from the Expectation Confirmation Model are supported. To separately test the mediating effects of perceived usefulness, expectation confirmation, and satisfaction in the relationships between course characteristics, platform features, and continued usage intention, this study employed the bias-corrected nonparametric percentile Bootstrap method, and the results are presented in Table 7.

**Table 6:** Results of hypothesis testing (N = 1592).

	EC	PU	SA	CI
PSQ	0.378***	0.432***		0.392***
PAV	0.353***	0.374***		0.305***
PI	0.314***	0.333***		0.243***
EC		0.755***	0.674***	
PU			0.493***	
SA				0.587***
*R*^2^	0.235	0.326	0.452	0.265
F	3.842***	5.426***	6.524***	4.985***

*EC* Confirmation of expectations, *PU* perceived usefulness, *SA* satisfaction, *CI* continuance intention, *PI* perceived interactivity, *PAV* perceived added value, *PSQ* platform service quality, The diagonal bold word is square root of average variance extracted (AVE). *p < 0.05, **p < 0.01, ***p < 0.001.

**Table 7:** The effect values and confidence intervals of the paths (N = 1592).

Variables	Effect values	Bootstrapped standard error	Bootstrapped confidence interval lower bound	Bootstrapped confidence interval upper bound
PI → PU → SA → CI	0.0774	0.0201	0.0453	0.1238
PI → EC → SA → CI	0.0983	0.0256	0.0604	0.1548
PAV → PU → SA → CI	0.0724	0.0182	0.0433	0.1154
PAV → EC → SA → CI	0.0963	0.0243	0.0612	0.1485
PSQ → PU → SA → CI	0.0742	0.0198	0.0395	0.1104
PSQ → EC → SA → CI	0.0874	0.0231	0.0515	0.1436

*EC* Confirmation of expectations, *PU* perceived usefulness, *SA* satisfaction, *CI* continuance intention, *PI* perceived interactivity, *PAV* perceived added value, *PSQ* platform service quality, The diagonal bold word is square root of average variance extracted (AVE). *p < 0.05, **p < 0.01, ***p < 0.001*.*

Results indicate that the mediating effects of course characteristics, platform features, and continued usage intention are significant. In the mediation path **PSQ → EC → SA → CI**, the effect value is 0.0874, with a 95% Bootstrap confidence interval ranging from 0.0515 to 0.1436, excluding 0. This implies that expectation confirmation and satisfaction play a significant mediating role in the relationship between platform service quality and continued usage intention, supporting ***H9-1***. In the mediation path **PSQ → PU → SA → CI**, the effect value is 0.0742, with a 95% Bootstrap confidence interval ranging from 0.0395 to 0.1104, excluding 0. This suggests that perceived usefulness and satisfaction significantly mediate the relationship between platform service quality and continued usage intention, supporting ***H9-2***. In the mediation path **PI → EC → SA → CI**, the effect value is 0.0983, with a 95% Bootstrap confidence interval ranging from 0.0604 to 0.1548, excluding 0. This indicates that expectation confirmation and satisfaction play a significant mediating role in the relationship between perceived interaction and continued usage intention, supporting ***H10-1–1***. In the mediation path **PI → PU → SA → CI**, the effect value is 0.0774, with a 95% Bootstrap confidence interval ranging from 0.0453 to 0.1238, excluding 0. This shows that perceived usefulness and satisfaction significantly mediate the relationship between perceived interaction and continued usage intention, supporting ***H10-1***. In the mediation path **PAV → EC → SA → CI**, the effect value is 0.0963, with a 95% Bootstrap confidence interval ranging from 0.0612 to 0.1485, excluding 0. This indicates that expectation confirmation and satisfaction play a significant mediating role in the relationship between perceived added value and continued usage intention, supporting ***H10-2***. In the mediation path **PAV → PU → SA → CI**, the effect value is 0.0724, with a 95% Bootstrap confidence interval ranging from 0.0433 to 0.1154, excluding 0. This suggests that perceived usefulness and satisfaction significantly mediate the relationship between perceived added value and continued usage intention, supporting ***H10***.

## Discussion

This study delves into the factors influencing the continued usage intention of adult learners on online education platforms, focusing on both platform features and course characteristics^56^. Beginning with platform features, we examined the impact of platform service quality on user experience. The research revealed that service quality has a direct relationship with users' expectation confirmation and perceived usefulness of the platform^1,98^. This aligns with previous research findings, emphasizing adult learners' expectations for high-quality services, including effectiveness and promptness, when using online education platforms. It also corroborates the Information Success Model constructed by Delone et al.^31^, where service quality is identified as a crucial factor significantly affecting satisfaction and usefulness^41^. The study results indicate that enhancing platform service quality will directly strengthen adult learners' expectation confirmation and perceived usefulness, fostering a more positive online learning habit among adult learners.

Furthermore, concerning course characteristics, we gauged the appeal of courses through perceived interactivity and perceived added value^99^. The research results demonstrate a significant positive relationship between adult learners' positive perceptions of course characteristics and their expectation confirmation, perceived usefulness, satisfaction, and ultimately, their intention to continue using the platform^99^. This validates the importance of interactivity and added value in previous research, particularly in the context of online education^34^. Adult learners anticipate courses to have robust interactivity, fostering positive interactions among students and addressing issues promptly, thereby enhancing adult learners' learning expectations^44^. Simultaneously, adult learners positively evaluate the added value provided by the courses, further reinforcing their perception of the course's value and subsequently increasing expectation confirmation and satisfaction.

Adult learners' intention for continuous usage is a complex process influenced by multiple factors related to platform and course characteristics^57,100,101^. Improvements in platform service quality and course features have a positive impact on adult learners' expectation confirmation, perceived usefulness, and satisfaction, ultimately encouraging adult learners to actively choose to continue using the online education platform^58^. Furthermore, through the analysis of mediating effects, we have identified that expectation confirmation and satisfaction play a chain-mediated role between platform service quality and adult learners' intention for continuous usage^44^. This suggests that enhancing platform service quality will strengthen users' intention for continuous usage by elevating expectation confirmation and satisfaction^57^. Therefore, educational platforms, in enhancing adult learners experience, should not only focus on improving platform service quality but also prioritize optimizing course characteristics to comprehensively enhance adult learners' online learning experience and loyalty.

This study reveals that learners' expectation confirmation and online learning engagement significantly influence learning satisfaction and perceived outcomes across different educational contexts. In formal education, enhancing teaching quality and learner engagement can boost learning outcomes^102^. In corporate training, practical, work-related content and high-quality teaching can heighten satisfaction and perceived outcomes^103^. In informal learning, catering to individual needs and providing a flexible learning environment can significantly enhance satisfaction and learning outcomes^58^. These insights offer valuable strategies for boosting learning satisfaction and perceived outcomes in diverse learning environments.

In the discussion section, we can further explore how emerging technologies, particularly Artificial Intelligence (AI), could potentially influence the relationships outlined in our hypotheses^104,105^. The advent of AI offers new possibilities for online education, especially in enhancing the quality of teaching services^105,106^. For instance, AI can be used to develop intelligent tutoring systems that provide personalized learning experiences based on learners' styles and needs, potentially enhancing their expectation confirmation and, consequently, their learning satisfaction and perceived learning outcomes^107^. However, the application of AI in online education also presents challenges, such as ensuring the fairness and transparency of AI systems and protecting learners' privacy.

## Implications

### Implications for theory

In terms of theoretical implications, this study offers fresh insights into the learning experiences of adult learners on online education platforms. The findings reveal that the confirmation of adult learners' expectations positively impacts both their perceived usefulness and satisfaction. This underscores the pivotal role of user expectation confirmation in the online learning experience, highlighting the correlation between expectations and experiences^100^. Simultaneously, the research results provide profound insights into the psychological gap in the adult learners' experience of online education platforms, offering a theoretical basis for understanding the relationship between adult learners' expectations and actual experiences.

However, to further enhance the value of the research, it is suggested to integrate new context-specific structures and novel theories to improve the parsimony and novelty of the research. For instance, exploring other potential factors in the online education environment, such as community atmosphere and interaction quality, and how they influence adult learners' expectation confirmation and satisfaction could be beneficial^99^. Additionally, adopting novel theoretical perspectives, such as self-determination theory, can provide a new viewpoint for understanding the motivations and behaviors of adult learners in online learning^101^.

### Implications for practice

This research offer practical guidance for online education providers, emphasizing the need to enhance platform service quality and course features to meet adult learners' expectations and perceived usefulness. The study provides valuable insights for formulating strategies to improve user satisfaction and foster continuous usage intention among adult learners in the competitive online education market.

#### A truthful promotional strategy

The research results suggest that online education platforms should adhere to a truthful approach in their promotion and avoid exaggerated claims. This provides practical guidance for operations, helping the platform establish an authentic and trustworthy image in advertising and marketing^52^. This approach reduces the potential psychological gaps that users might experience after use, thereby enhancing overall user satisfaction. This strategy is universally applicable, not only in a formal education environment but also in corporate training and informal learning settings.

#### Emphasizing the utility in promotion

Online education platforms should highlight their strengths, features, and content quality to help users profoundly understand the platform's utility^44^. This provides direction for the platform in advertising and promotion, aiding users in gaining a more comprehensive understanding of the platform's value, thereby increasing perceived utility and overall satisfaction^12^. This approach is applicable across various educational settings, benefiting formal education, corporate training, and informal learning alike.

#### Enhancing service quality

The study reveals a significant positive impact of platform service quality on user expectation confirmation and perceived utility^57^. Therefore, platforms should invest in professional training for customer service to improve service quality, aiming to enhance adult learners' expectations and subsequently elevate perceived utility^52^. This practical recommendation provides online education platforms with actionable insights to improve adult learners' experience and contributes to establishing a solid foundation for user satisfaction^5^. Improving service quality is crucial in all educational environments, whether it's formal education, corporate training, or informal learning.

#### Optimizing course experience

By emphasizing perceived added value and interactivity, platforms can enhance adult learners' satisfaction with course quality^53^. To achieve this, platforms can offer additional services during adult learners' engagement and establish communication channels, enabling adult learners to better experience the utility of the online education platform^103^. This provides a practical and feasible approach for platforms to optimize the course experience and increase user willingness to continue using the platform^58^. This approach has application value in different educational settings and can help improve the course experience in formal education, corporate training, and informal learning.

## Limitations and prospects

This study has made notable discoveries about adult learners' sustained intention to utilize online education platforms, but it has limitations. Firstly, the research mainly hinged on adult education students from a single university, potentially limiting the results' broad applicability due to sample specificity. Future research could enhance the findings' external validity by expanding the sample size and incorporating more diverse user groups. Secondly, despite a comprehensive research model, additional latent variables influencing adult learners' continued intention to use online platforms might have been overlooked. Future studies could explore other potential factors for a more comprehensive understanding of the decision-making process when adult learners opt to persist with online education platforms.

Additionally, this study predominantly employs quantitative research methods, leveraging survey data collection. Future research could contemplate integrating more qualitative research methods, like in-depth interviews or observations, to attain a more comprehensive grasp of adult learners' behavioral motivations and experiences^108^. Future research should also advocate for the use of mixed methods or longitudinal studies to empirically substantiate the proposed hypotheses across various types of online education platforms and diverse adult learner populations.

Subsequent research could incorporate negative outcomes, as current research mainly focuses on positive ones. Considering the potential negative impact of high expectations or poor service quality on user satisfaction and continuance intention can provide a more comprehensive understanding of the online learning experience. The rapid development of online education technology necessitates future research to include factors associated with technological advancement (for example, personalized learning driven by artificial intelligence) and their impact on the learning experience.

The upcoming research has the potential to introduce negative outcomes, given that current studies primarily concentrate on positive results. Taking into account the potential negative influence of high expectations or subpar service quality on user satisfaction and sustained intention can offer a more comprehensive comprehension of the online learning experience. Future research should consider the speedy evolution of online education technology, integrating factors related to technological progress (for instance, AI-driven personalized education) and their repercussions on the learning experience can be highly valuable.

Looking ahead, researchers can dedicate efforts to further deepen the investigation into adult learners' continuous intention to use online education platforms, overcoming current study limitations, and continually enhancing the understanding of the mechanisms behind adult learners behaviors.

## Conclusions

Based on the Expectation Confirmation Theory and considering the characteristics of online education platforms, this study constructs a research model by focusing on two crucial variables—expectation confirmation and perceived usefulness—and their influencing factors: platform service quality and course service quality. The results indicate the following key findings: (1) Satisfaction is a critical factor influencing adult learners' continued usage of the platform. (2) Adult learners' perceived usefulness affects satisfaction, and the degree of expectation confirmation significantly influences both perceived usefulness and satisfaction. (3) Platform service quality impacts expectation confirmation and plays an essential role in perceived usefulness. (4) Perceived added value and perceived interactivity of course service quality significantly influence expectation confirmation and also play a crucial role in perceived usefulness. (5) Perceived usefulness, expectation confirmation, and satisfaction serve as significant mediators in the relationship between platform features and the intention to continue usage. (6) Perceived usefulness, expectation confirmation, and satisfaction act as significant mediators in the relationship between course features and the intention to continue usage. In summary, these findings shed light on the factors influencing users' continued usage of online education platforms, providing valuable insights for platform operators to enhance user experience and satisfaction.

## Data Availability

The datasets generated and analysed during the current study are available in the Zenodo repository, 10.5281/zenodo.10584056.
